# Water properties under nano-scale confinement

**DOI:** 10.1038/s41598-019-44651-z

**Published:** 2019-06-03

**Authors:** Andrew W. Knight, Nikolai G. Kalugin, Eric Coker, Anastasia G. Ilgen

**Affiliations:** 1 Geochemistry Department, Sandia National Laboratories, 1515 Eubank Blvd SE, Albuquerque, NM 87185-0754 United States; 2 Department of Materials and Metallurgical Engineering, New Mexico Tech, 801 LeRoy Place, Socorro, NM 87801 United States; 3 Applied Optical and Plasma Science Department, Sandia National Laboratories, 1515 Eubank Blvd SE, Albuquerque, NM 87185-0754 United States

**Keywords:** Geochemistry, Pollution remediation

## Abstract

Water is the universal solvent and plays a critical role in all known geological and biological processes. Confining water in nano-scale domains, as encountered in sedimentary rocks, in biological, and in engineered systems, leads to the deviations in water’s physicochemical properties relative to those measured for the non-confined phase. In our comprehensive analysis, we demonstrate that nano-scale confinement leads to the decrease in the melting/freezing point temperature, density, and surface tension of confined water. With increasing degree of spatial confinement the population of networked water, as evidenced by alterations in the O-H stretching modes, increases. These analyses were performed on two groups of mesoporous silica materials, which allows to separate pore size effects from surface chemistry effects. The observed systematic effects of nano-scale confinement on the physical properties of water are driven by alterations to water’s hydrogen-bonding network—influenced by water interactions with the silica surface — and has implications for how we understand the chemical and physical properties of liquids confined in porous materials.

## Introduction

The physicochemical properties of water are controlled by the hydrogen- (H) bonding network, which is comprised of intermolecular interactions of water molecules^1^. These H-bonding interactions are dynamic and, due to the molecular shape and dipole moment of water, each molecule can form up to four direct H-bonds^2^.

Water existing in geological, biological, and engineered environments is often present trapped in nanometer-scale cavities, or adsorbed as nm-scale water films. Examples of these confined systems include pores of zeolites, clay mineral interlayers, voids in concrete, and nano-scale pores and channels of sedimentary rocks and soils^3,4^. Additionally, confined water is common in biological systems and water purification membranes^4^. Spatial confinement leads to deviations in the thermodynamic and physical properties of water, compared to those observed in the bulk non-confined phase. Recent computational and experimental work have demonstrated deviations in the properties of water inside nano-scale pores^5^. These physical changes include decreases in the freezing point, density, surface tension, and dielectric constant of water^6–9^. It was also shown that H-bonding networks undergo measurable changes when water is confined^5,10^. These observations demonstrate shifts in the vibrational features of water, induced by nano-scale confinement, and conclude that both the degree of confinement—e.g. size of the domain—as well as the surface structure and reactivity play a pivotal role in influencing the H-bonding network.

Emergent behavior of water under nano-scale confinement arises when the ratio of water interacting with the confining surface approaches unity with the remaining bulk water. At this point, the nano-scale confinement effect on the H-bonding network becomes increasingly apparent^3^. Current literature reports emergent nano-scale confined water behavior occurring in pores over a vast range of pore diameters. Where some research shows that in large nano-scale pores, with 50 nm^4^ to 320 nm^5^ diameter, these effects are observed, while other studies suggest that observable changes are limited to confined systems with a pore diameter less than 10 nm – as reported for porous silica glass^1,11^ and reverse micelles^10,12^. This discrepancy may be due to the fact that, in addition to the pore-size effects, the nano-scale confinement-induced alterations to water’s H-bonding network is dependent upon the reactive surface sites, surface charge, and other characteristics of the confining surface.

Therefore, there exists a critical need to understand nano-scale confinement effects on the physicochemical properties of confined water^13^. Closing the current knowledge gap is essential for applications in geochemical and biological sciences^12^, condensed matter (*i.e*. micro-emulsions and reverse micelles)^10,12^, catalysis^14^, membrane separation science^15^, and nano-fluidics^16^. Motivated by previous research, our work simultaneously interrogates the physicochemical and vibrational properties of water residing inside nano-scale pores for two classes of mesoporous silicas, with pore diameters ranging from 8 to 2 nm, as well as one control sample (previously investigated by Takei *et al*.^7^), to systematically quantify the impacts of nano-scale confinement on H-bonding interactions of water confined inside silica mesopores. Mesoporous silicas are excellent materials to explore the effects of nano-scale confinement on water as they offer experimental advantages, including: (*i*) the surface of amorphous silica has silanol (Si-OH) functional groups, which act as a proxy for silicate minerals^17^, and (*ii*) the pore diameter distribution is narrow, allowing for a targeted study to isolate the effect of decreasing pore size, while maintaining a constant surface reactivity. To our knowledge, this is first simultaneous evaluation of the physical *and* vibrational properties of confined water in a systematic matrix of mesoporous materials, which provides the framework for the most comprehensive analysis of the impact that nano-scale confinement has on water.

### Hydrogen-Bonding Networks and Physical Properties of Nano-scale Confined Water

Two classes of mesoporous silicas were used in our experimental work: (1) Santa Barbara amorphous (SBA-15), which has long channel pores with hexagonal cross-section and particles that are <150 µm, (2) mesoporous silica *ms*-silica, with random pore geometry with particles that are about 3 µm in diameter, and (3) Mobil Composition of Matter No. 41(MCM-41), with hexagonal channel pores and rod-like particles. The surface properties of mesoporous silica materials, used to confine water SBA-15-8, SBA-15-6, SBA-15-4, *ms*-silica-4, *ms*-silica-2 and MCM-41, analyzed by thermoanalysis and Brunauer-Emmett-Teller (BET) nitrogen (N_2_) adsorption, are summarized in Table 1. These properties include average pore diameter, BET surface area, pore volume, and surface hydroxyl group (OH^−^) density. We observed a slight and systematic decrease in the OH^−^ group density, with decreasing pore size for SBA-15 series (Fig. S1, Supporting Information), while the OH^−^ density increased with decreasing pore size for *ms*-silicas.

**Table 1:** Physical properties of silica materials including, pore size, BET surface area, pore volume, OH group density, and reference.

Material	Pore diameter (nm)	BET Surface Area (m^2^/g)	Pore Volume (cm^3^/g)	–OH^−^ density (–OH/nm^2^)	Reference
SBA-15-8	7.0 ± 0.3	661 ± 5	1.21 ± 0.03	1.8 ± 0.2	Knight *et al*.^44^
SBA-15-6	5.2 ± 0.2	603 ± 16	0.87± 0.03	1.9 ± 0.2	Knight *et al*.^44^
SBA-15-4	4.4 ± 0.1	580 ± 13	0.67 ± 0.04	2.3 ± 0.2	Knight *et al*.^44^
*ms-silica-4*	6.6 ± 0.1	253 ± 3	0.45 ± 0.03	2.6 ± 0.3	This study
*ms-silica-2*	2.2 ± 0.2	1090 ± 19	0.50 ± 0.02	1.1 ± 0.2	This study
MCM-41	1.55^7^	861^7^	0.865^7^	0.59 ± 0.3^†^	Takei *et al*.^7^, ^†^This study

**Figure 1:**
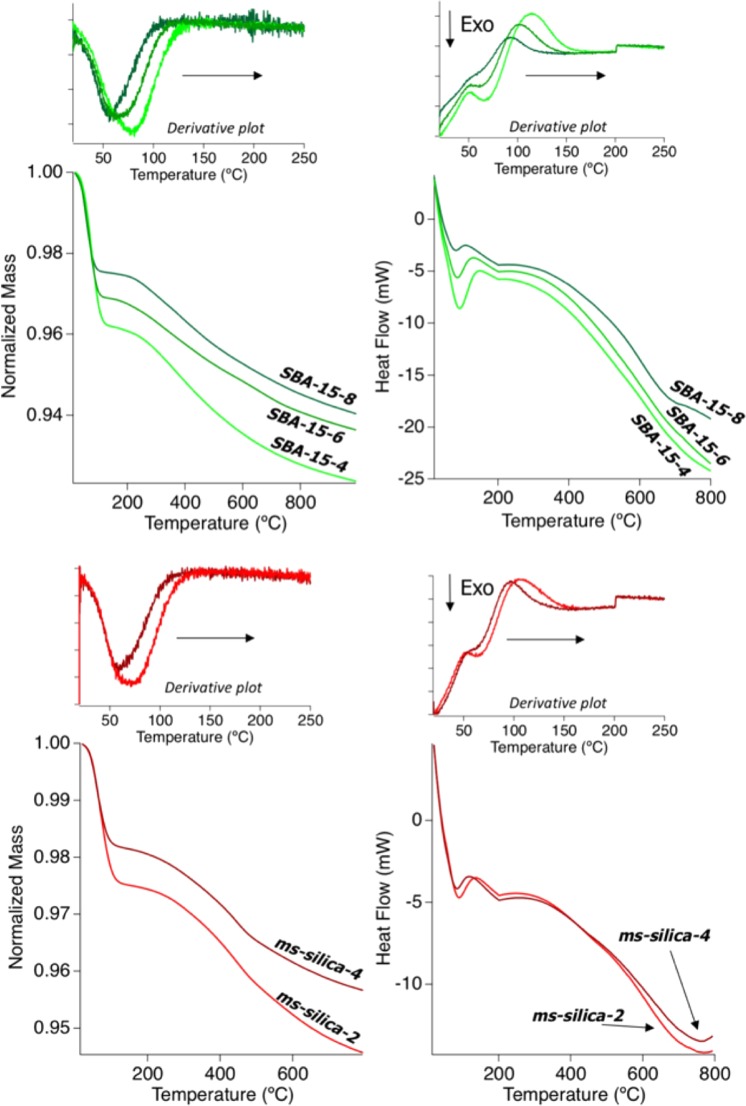
Thermogravimetric analysis and direct scanning calorimetry of silica samples grouped by SBA-15 samples and *ms*-silica samples shown with their derivative plots, respectively. The arrow indicates the trend with decreasing pore diameter.

**Figure 2:**
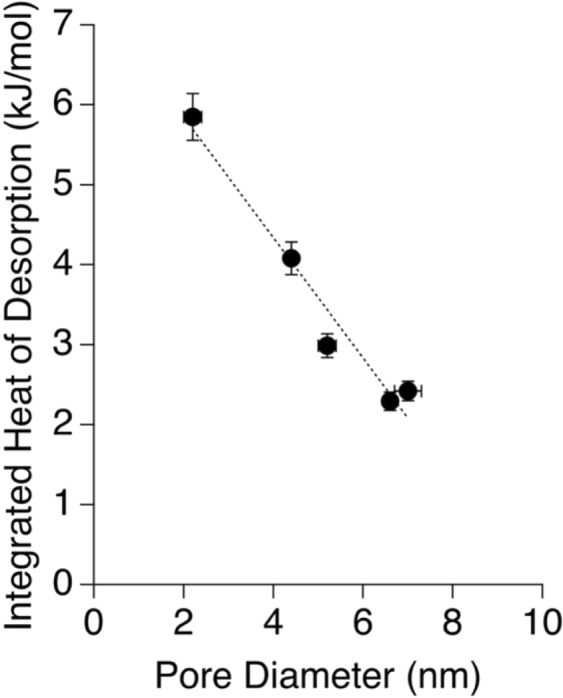
Integrated heat of water desorption (kJ/mol), calculated from integrating the differential scanning calorimetry, versus the silica pore diameter.

Thermal analyses, including thermogravimetric (TGA) and differential scanning calorimetry (DSC), demonstrate that the pore diameter controls the mean temperature of water desorption (Fig. 1). The desorption of physisorbed water takes place up to 200 °C (473.15 K), while at higher temperatures mass loss is due to de-hydroxylation of silica surfaces^18^. We observed that with decrease in the pore diameter, the water desorption temperature systematically increases, and the maximum rate of water loss increases as well. Likewise, the total mass of physisorbed water increases with decreasing pore size: 2.6% for SBA-15-8, 4.0% for SBA-15-4, 2.0% for *ms*-silica-4, and 2.6% for *ms*-silica-2. This suggests that there is an increased affinity of water molecules for silica surface as pore size decreases. For SBA-15 samples, the increased water incorporation could be correlated to the OH^−^ group densities, however for *ms*-silica samples the differences may be attributed to the differences in the surface area (Table 1). The integrated heat associated with water desorption has a negative linear correlation with pore size, where water desorption is more exothermic as pore diameter decreases (Fig. 2). The total heat required to desorb water, along with the temperature when the maximum exothermic heat flow rate occurs, are pore size dependent. These results suggest an increased population of structured water—water with increased degree of H-bonding—because more energy is required to desorb water from the surface or the pores^19^. Furthermore, Fig. 2 shows an increased heat flow (around 100 °C, 373.15 K) at increased temperatures directly proportional to pore size.

**Figure 3:**
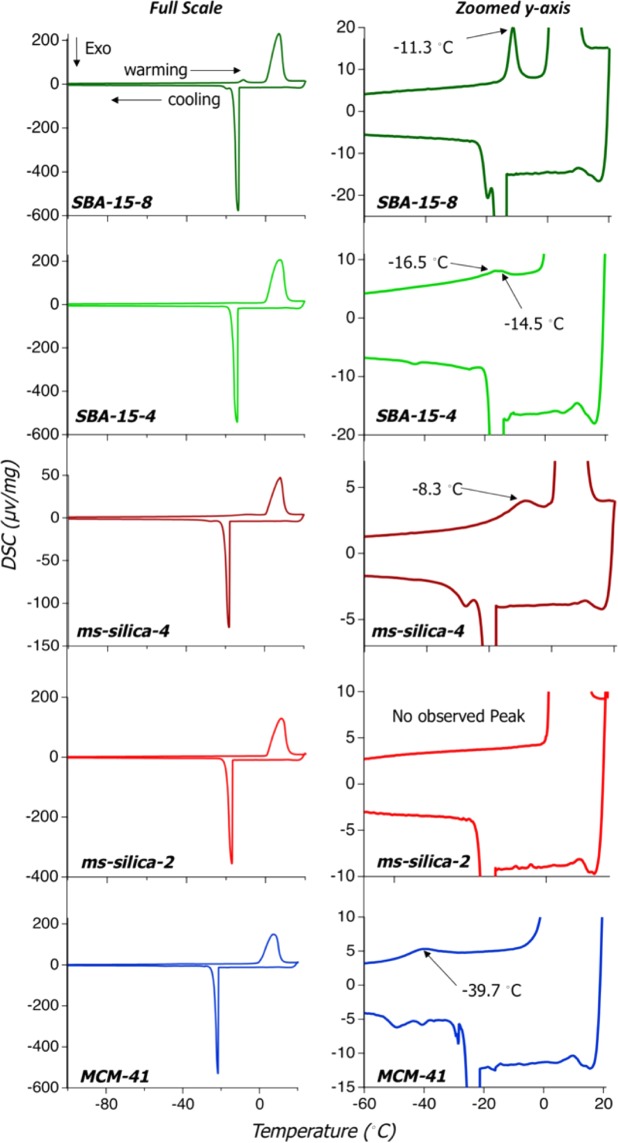
Melting point temperatures of each silica material SBA-15-8, SBA-15-4, *ms*-silica-4, *ms*-silica-2, and MCM-41. For each, a full spectrum analysis is shown, where the large features are attributed to bulk water freezing and melting. Additionally, a zoomed in y-axis view showing the freezing and melting of confined water, where melting temperatures are annotated.

**Figure 4:**
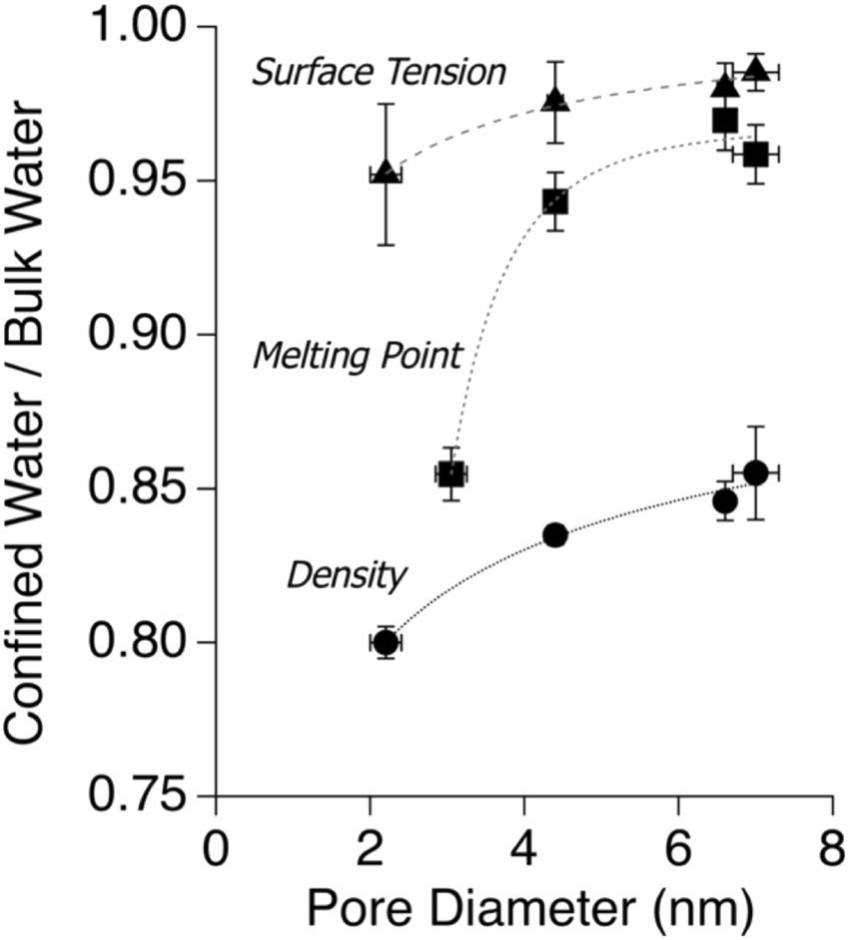
The calculated relative surface tension and density, determined by BET N_2_ and water adsorption studies, along with relative melting point temperature, determined by differential scanning calorimetry, versus silica pore radius.

The melting point temperature for water residing inside the nano-scale pores decreases with nano-scale confinement (Fig. 3). The melting of the bulk water is observed by the broad feature around 0 °C (273.15 K), whereas the melting points of the confined water are shown in the zoomed y-axis insert of each figure panel. The small features prior to the melting of bulk water indicate that the water confined in the pores melts at a lower temperature, and that the melting point temperature is dependent upon the pore diameter. This observation is consistent with large body of work, including NMR cryoporometry, centered around understanding the effects of pore size and shape on melting and freezing temperatures^20–25^. The Gibbs-Thomson equation has been used to determine the melting point depression for a small, isolated spherical crystal based upon pore size and surface properties^22,23,25^. Our results agree with those observed by Jähnert *et al*. 2008 investigated the freezing and melting points of H_2_O and D_2_O in MCM-41 silica materials with pore sizes ranging from 2.5 nm to 4.4 nm, and Rottreau et. al., 2018 investigated melting point depression of SBA-15, SBA-16 and KIT-6 *via* cryoporometry^21,23^. Our results demonstrate a 5% decrease in the melting point temperature of SBA-15-8, up to a 15% decrease in the melting point temperature for MCM-41 - Rotteau et. al. 2018 observed ~3%, ~4%, and ~8% decrease in melting point for 8 nm, 4 nm and 2 nm silica pores using the Gibb-Thomson relationship^23^. This trend is confirmed by the results reported by Jähert *et al*. 2008 and as pore size increases, the observed melting point temperatures approach that of bulk water, shown in Fig. 4. Further, we observed no melting point temperature in the smallest pores 2.2 nm (*ms-*silica-2), similar to the smallest pores analyzed by Jähnert *et al*. 2008, 2.5 nm. From this observation, Jähnert *et al*. 2008 concluded that water does not undergo a first-order phase transition in domains smaller than 2.5 nm^21^.

Water and N_2_ adsorption on mesoporous silica materials was measured to estimate the density and surface tension of water inside silica pores (Fig. S2). This method was adapted from Takei *et al*. 2000, and described in detail in the Methods Section^7^. The density and surface tension of water decreased as the pore diameter decreased. Our findings suggest a 15–20% decrease in the density of water due to confinement in pores ranging in size from 7.7 to 2.2 nm. This is consistent with reported literature, which suggested that both the density and surface tension of water confined inside nano-scale pores is less than in the bulk phase, and becomes equal to the bulk values when pores have a diameter of 10 nm or larger^7,26,27^. This phenomenon is due to the existence of electrical double-layer at an aqueous-solid interface inside the pores. When confining surfaces are sufficiently close, the electrical double-layers start to overlap, affecting the water properties inside the pores. The first layer of adsorbed water is physisorbed to the pore surface, through interactions with the surface hydroxyl groups, then the subsequent layers are affected by the orientation and configuration of the first layer of adsorbed water up to about 3–5 nm from the surface^7,27,28^. Our data suggests that the density and surface tension may achieve the bulk-phase values at a pore diameter larger than 10 nm. This slight discrepancy could arise from differences in the surfaces and materials used, as well as the composition of the aqueous phase. Our studies investigated SBA-15, *ms-*silica, and MCM-41, whereas Takei *et al*. 2000 analyzed MCM-41 and porous silica glass (PSG). One possible explanation is that the PSG materials contains less surface hydroxyl groups and therefore could be more hydrophobic than SBA-15 and *ms-*silica resulting in less water entering the pores^7^.

Changes to the density and surface tension of water are likely resulting from a decrease in the intermolecular forces^7^. Takei *et al*. 2000 hypothesized that the decrease in surface tension and density arose from (*i*) a decrease in the coordination number (CN), (*ii*) an increase in the distance between nearest neighbor molecules, and/or (*iii*) a change in the O-H•••H angles. The authors concluded that the H-bonding interactions, and therefore hydrophilicity, is important for the observed decrease the surface tension, by comparing H_2_O and carbon tetrachloride CCl_4_ confined in a silica pore^7^. Other studies state that the surface tension of a liquid deviates from bulk water below the mean radius of curvature of the meniscus, which occurs at around 10 nm, and that the surface tension decreases by 30% when the radius is 2 nm^29^. This is further supported by calculations investigating various liquids, including argon (Ar), N_2_, benzene (C_6_H_6_), hexane (C_6_H_12_), and H_2_O, and observing a change in the surface tension when pores are smaller than 10 nm, with an observed increase corresponding with concavity and a decrease with convexity of liquid surfaces^30^.

The CN of water and density in confinement have been the topic of many manuscripts. Takei *et al*. 2000 postulate that the decrease in water density can be attributed to a decrease in the CN toward CN = 4, similar to ice, from CN = 4.4, as seen in bulk water^7,31^. Similar conclusions have been made by Iiayama *et al*. 1995, having shown through an X-ray diffraction assessment of adsorbed water confined in carbon nanopores with a 1.3 nm diameter, the number of nearest neighbors decreased, while long range ordering increased^26^. Yet, Wernet *et al*. 2004, *via* X-ray adsorption spectroscopy, suggest that bulk ice (Ih) is tetrahedrally coordinated, but the exact H-bonding environment is still questioned, citing hypotheses that a large fraction of the water molecules near the surface have one free O-H group (CN ≤ 3)^32^. Hou *et al*. 2016, in a molecular dynamic analysis of aqueous NaCl in nano-pores of portlandite, saw a different trend mentioning that the CN of a single water molecule is on average 4.9 at 0.12 nm, and increase to 5.1 before returning to bulk water CN of 4.4. This result suggests that surface interactions influence the CN of water by orienting the surface water, and this influence can be exaggerated by confinement^31^. We will continue the conversation regarding the CN of confined water later in this manuscript when discussing vibrational properties of water inside nano-scale silica pores.

**Figure 5:**
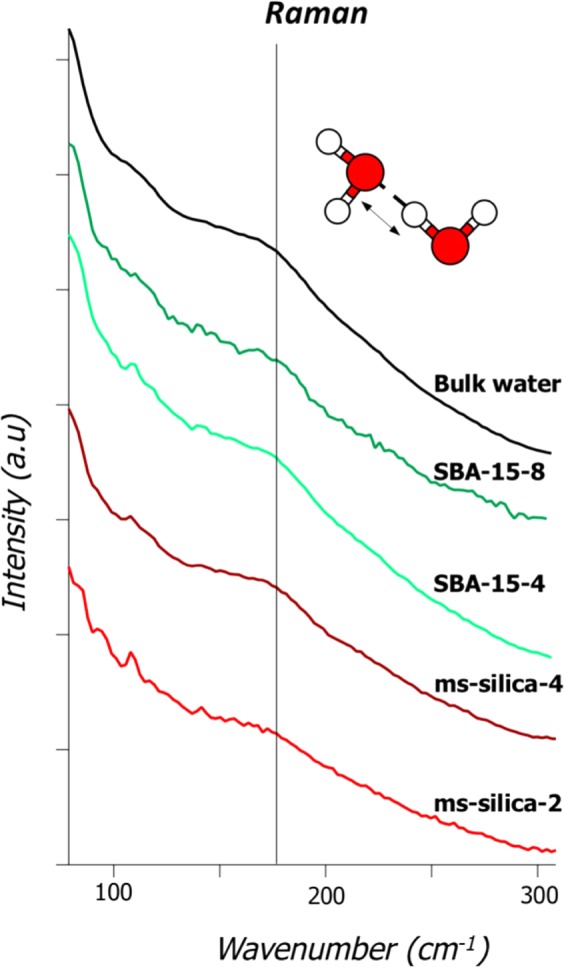
Raman spectra showing the connectivity band of free bulk water, SBA-15-8, SBA-15-4, *ms*-silica-4, and *ms*-silica-2.

To gain a detailed understanding of the molecular interactions of water confined in mesoporous silica, we probed changes to the H-bonding vibrational modes by Raman scattering and attenuated total reflectance Fourier transform infrared spectrometry (ATR-FTIR). The connectivity band, typically located around 170–180 cm^−1^, results from longitudinal motion of H atoms along a H-bonding axis, O-H•••H^5,33^. This provides an indication of the extent of H-boding occurring between neighboring water molecules, where higher frequency vibrations suggest stronger H-bonding between water molecules (Fig. 5, Table 2)^5^. All samples with confined water (SBA-15-8, SBA-15-4, *ms-*silica-4 and *ms*-silica-2), had a smaller full width at half maximum (FWHM) compared to bulk water, where the FWHM of bulk water was 35 cm^−1^ and for confined water it ranges between 24 to 26 cm^−1^. Furthermore, a blue shift was observed as pore size decrease, suggesting that the H-bonding network is higher in energy. The peak position of the connectivity band of bulk water was centered around 173 cm^−1^, where SBA-15 samples increased from 172 cm^−1^ for 8 nm pore to 177 cm^−1^ for the 4 nm pore, and from 174 cm^−1^ for *ms-*silica-4 to 176 cm^−1^ for *ms*-silica-2. The blue shift became more pronounced with an increasing degree of confinement. Le Caër *et al*. 2011 demonstrated both peak narrowing and blue peak shift of connectivity band using ATR-FTIR^5^. The peak narrowing was said to be related to a higher proportion of well-structured H-bonds at the interface or pore walls, and the blue shift may be caused by confinement effects leading to increased H-bonding interactions between water molecules, or increased ordering^5^. Another explanation for the blue shift in the vibrational frequency is a change in distribution of O-O distances of confined water, which have been measured by changes by IR as well as by neutron spectroscopy experiments^5,13,34^. One notable difference between our Raman results and IR data reported by Le Caër *et al*. 2011, is the magnitude of the shift of confined water compared to bulk water. We observed a blue shift in a few wavenumbers, while Le Caër *et al*. 2011 noted shifts by nearly 20%, which was noted to be a consequence of averaging, and that the connectivity band is composed of various types of H-bonding exchanging rapidly^4^. *Ab Initio* molecular dynamics (AIMD) suggest that the 0–200 cm^−1^ range is dominated by movement extending over just a few water molecules, and the lifetimes of the H-bonding interactions are on the pico-second time scale^4,34^.

**Table 2:** Raman peak positions and FWHM of the connectivity band.

Raman		
Material	Position (cm^−1^)	FWHM (cm^−1^)
Bulk water	173	35
SBA-15-8	172	24
SBA-15-4	177	24
*ms*-Silica-4	174	25
*ms*-Silica -2	176	26

The rotational motion and bending mode of confined water were evaluated, however no conclusive evidence of nano-scale confinement effects was observed in these vibrational regions. Further discussion of this data is available in the Supplemental Information and Figs S3, S4 and Tables S1, S2.

**Figure 6:**
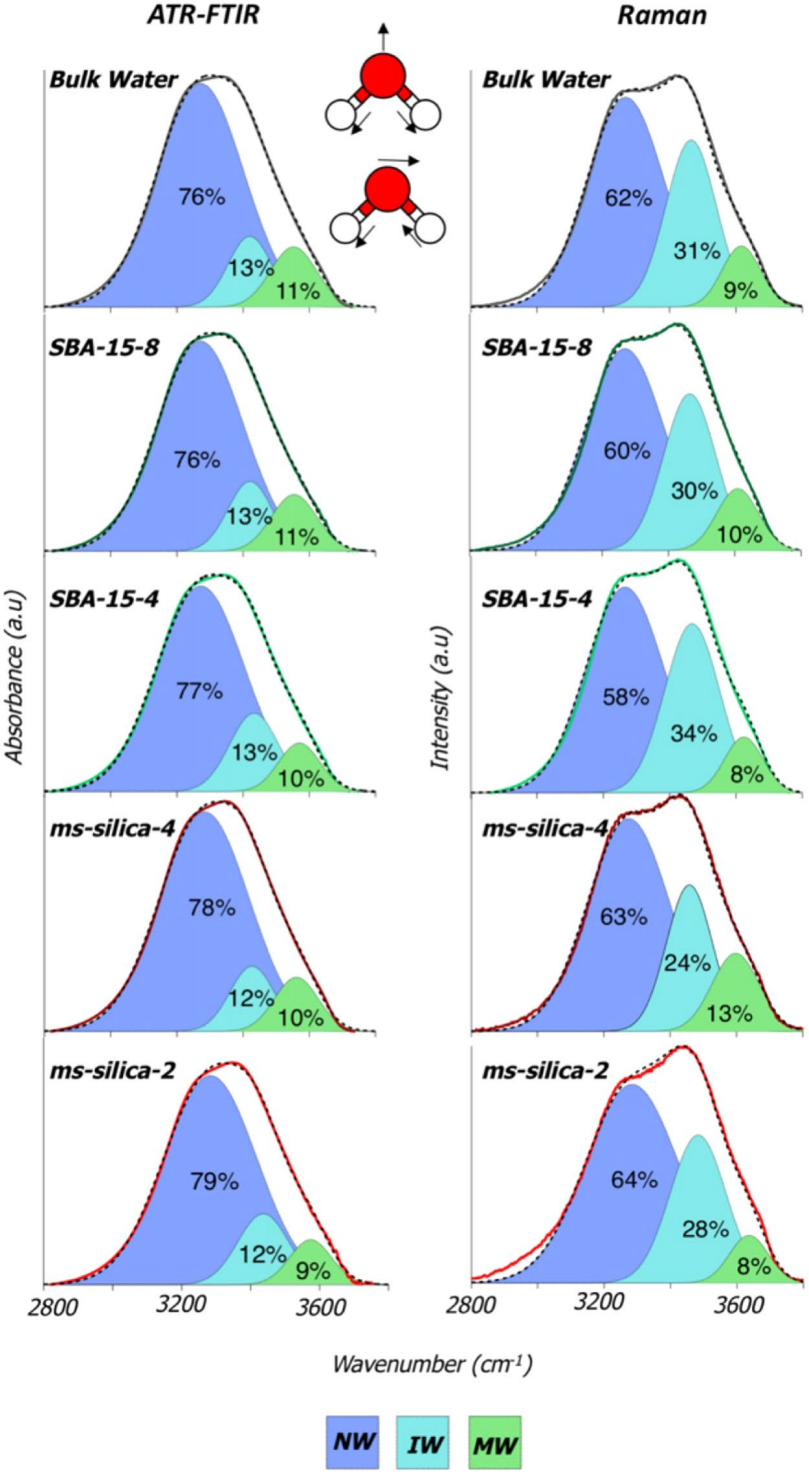
ATR-FTIR and Raman spectra showing the O-H stretching band of free bulk water, SBA-15-8, SBA-15-4, *ms*-silica-4, and *ms*-silica-2. The adsorption was fit with three features describing different H-bonding environments, specifically network water (NW), intermediate water (IW), and multimer water (MW). The dotted line in the ATR-FTIR data represents the global peak fit.

The water O-H stretch vibrational mode was also evaluated to probe the effects of nano-scale confinement as the O-H stretch provides direct intermolecular information sensitive to the strength of the H-bonding network^33^. We assumed that the O-H stretches resulted from water and not from SiO-H vibrations because of the analogous vibrations between the bulk water spectrum and the confined water. In general, as the number of intermolecular water-water interactions increases, the weaker the O-H stretching frequency, resulting in a red shift^5,33^. The O-H stretch of water confined in mesoporous silica is shown in Fig. 6 and summarized in Table 3. Previous research fit the O-H stretch region using 3–5 unique curves^1,5,10,11^. Our data was fit with three Gaussian curves, where the three curves, represent unique water populations, described as: network water (NW), intermediate water (IW), and multimer water (MW)^5,10,33,35,36^. Where NW corresponds to highly H-bonded water molecules, typically with a CN close to four, and oscillates near 3260 cm^−1^. As intermolecular water interactions decrease, IW water populations form, resulting in a vibrational peak around 3460 cm^−1^. Lastly, when water molecules interact with a small number of other water molecules (forming dimers or trimers), a blue shifted MW vibrational mode occurs and oscillates around 3610 cm^−1 ^^5^. The numerical percentages assigned to each of the three water populations represent qualitative estimates, and are used here to illustrate overall shifts in the average populations of water with increasing confinement.

**Table 3:** ATR-FTIR and Raman peak positions, FWHM, and percent contribution of each fit peak for the O-H stretching band.

Material	NW (Network Water)			IW (Intermediate Water)			MW (Multimer Water)			r^2^ of fit
	Position (cm^−1^)	FWHM (cm^−1^)	Percent (%)	Position (cm^−1^)	FWHM (cm^−1^)	Percent (%)	Position (cm^−1^)	FWHM (cm^−1^)	Percent (%)	
**ATR-FTIR**										
Bulk water	3272	308	76	3421	156	13	3554	163	11	0.997
SBA-15-8	3271	306	76	3420	156	13	3553	164	11	0.996
SBA-15-4	3474	315	77	3426	161	13	3560	158	10	0.997
*ms*-Silica-4	3279	315	78	3425	156	12	3558	159	10	0.992
*ms*-Silica -2	3285	320	79	3429	160	12	3564	153	9	0.994
**Raman**										
Bulk water	3267	137	60	3465	90	31	3616	69	9	0.998
SBA-15-8	3268	140	60	3464	93	30	3608	74	10	0.998
SBA-15-4	3265	139	58	3468	98	34	3624	68	8	0.997
*ms*-Silica-4	3277	146	63	3458	82	24	3597	84	13	0.996
*ms*-Silica -2	3283	165	64	3483	99	28	3636	69	8	0.993

We observed subtle changes in the water populations in both Raman and ATR-FTIR spectra. In the fit ATR-FTIR data, we see a systematic increase in NW and a slight decrease in IW and MW populations as pore diameter decreases. Likewise, we observed a systematic blue shift in the peak locations of NW, IW, and MW in confinement — consistent with previous work^5^. The largest shift occurs with *ms*-silica-2, where NW blue shifted by 16 cm^−1^, IW by 18 cm^−1^, and MW by 20 cm^−1^ in the Raman scattering. The magnitude of the population changes appears to be material dependent (i.e. surface area, pore morphology) as *ms*-silica-4 has a larger NW population than SBA-15-4, even though the pore size for *ms-*silica-4 is larger. Further, there was no difference between SBA-15-8 and bulk water, suggesting that a 7.7 nm pore diameter is large enough to not alter the O-H stretch to a measurable degree. In the Raman data, we observe an increased population of NW in *ms-*silica-4 over bulk water, and continued increase with *ms*-silica-2. However, with SBA-15-8 and SBA-15-4 the trend is not clear. We observed the same populations of water when bulk water is compared with SBA-15-8, which is consistent with our findings in ATR-FTIR; however, as pore size decreases - (SBA-15-4) we see a decrease in NW and MW while IW increased. Previous studies have shown a dramatic shift toward a larger population of NW as a result of nano-scale confinement^5,10,11^. Our data trends agree with previous observations, in that the most significant effects were observed in the smallest pores with diameter less than 4 nm^37–39^; however, some reports have shown an increase populations NW in pores are large as 320 nm^5^. Our vibrational data does not demonstrate a shift in water populations for pores greater than or equal to 7.7 nm. The global conclusion remains consistent; nano-scale confinement leads to an increased population of NW. However, the size of the pore diameter at which this phenomenon occurs still is not definitive.

Changes to the number of intermolecular water-water interactions in confinement results in anomalous chemical and physical properties, as well as shifts in bonding vibrational frequencies^1,2,5,13,33,40^. These observations have led to two bodies of work that discuss changes to the CN of water; where in (i) adsorption and physical measurements, the density and surface tension of water decrease with pore diameter, which previous research has attributed to a *decrease* in the CN of water from CN = 4.4 to 4.0 in a more ice-like confirmation^7,26,31^. On the other hand, (ii) vibrational studies, where populations of the highly-coordinated NW (CN~4) *increases* while populations of low coordinated MW (CN~1–2) *decreases* upon confinement^5^. Collectively, both sets of observations conclude that nano-scale confinement promotes more ice-like coordination environments (CN = 4); however, large populations of NW water suggests an *increase* in CN from bulk water, while lower densities suggest a *decrease* in CN. To reconcile this apparent discrepancy, previous work has proposed that a distribution of binding energies favors a continuous distribution of structures with CN = 3–6, and not a mixture model of liquid water–implying that water forms clusters in confinement^41^. Another study investigating water in SBA-3 pores by positron annihilation spectroscopy, observed distinct water-free void space and isles of water clusters centered around silanol groups, which the latter increased with increased pressure (or confinement) until capillary condensation occurred^42^. Therefore, we suspect that water in confinement is congregating around surface hydroxyl-groups to form islands of highly coordinated localized water regions. These islands form a discontinuous H-bonding network throughout the pore. This phenomenon would lead to increasing in vibrational modes associated with NW, through highly coordination water regions; however, the discontinuity of the H-bonding network as a whole would likely result in the observed changes in the physical properties (i.e. decrease density and surface tension).

For the first time we combined thermal analyses, water and gas adsorption, and vibrational spectroscopy methods to access the changes in physicochemical properties of water confined within nano-scale pores in two classes of mesoporous silicas. We quantified systematic changes in water properties in nano-scale confinement. As pore diameter decreases, the heat of desorption increase, while the density and surface tension decrease. The vibrational spectra indicate the changes to the to the H-bonding network are more pronounced as pore size decreases. Similar to temperature effects, water that exists in nano-scale confinement begins to behave more like ice in the examined mesoporous silica systems. These effects are most notable when pores are smaller than 5 nm, due to the increased influence of the surface water-silanol interactions that incorporate orientation and structure to the water molecules. We highlight that even though the re-structuring of H-bonding network due to nano-scale confinement is subtle, this re-structuring leads to dramatic decrease in the melting/freezing temperature of water, and measurable decrease in surface tension and density, as well as an increase in the water desorption enthalpy.

## Methods Section

### General

All gasses and cryogenic liquids used for BET surface area analysis were ultrapure quality grade (Matheson, Basking Ridge, NJ) including liquid and gaseous argon (Ar) and nitrogen (N_2_), along with helium (He) gas. Water used for these studies were Milli-Q H_2_O (Barnstead NANOpure Diamond, resistivity of 18.2 MΩ*cm, 0.2 μm filtered and UV irradiated) was used in the preparation of all solutions and suspensions.

All mesoporous materials were purchased from Sigma Aldrich (Sigma Aldrich, St. Louis, MO). These materials included two series of mesoporous silica; Santa Barbara Amorphous (SBA-15)^43^ including: SBA-15-8, SBA-15-6, SBA-15-4 with <150 μm particle size and hexagonally ordered cylindrical pores with diameters of 8 nm, 6 nm, and 4 nm; and *ms*-silica including: *ms-*silica-4 and *ms-*silica-2 with 3 μm spherical particles and disordered cylindrical pores with 4 nm and 2 nm pore diameters; lastly MCM-41 with 1.55 nm pores was used as a control to compare with Takei *et al*. (2000). Prior to analyses, each silica material was hydrated by one of two methods, (*i*) room temperature hydration or (*ii*) boiling. The room temperature hydration method was described previously^44^. Both methods resulted in nearly identical water adsorption and surface hydroxyl group densities. Briefly, approximately 600 mg of silica was added to centrifuge bottle with 200 mL of Milli-Q water and mixed on a shaker table (Orbital-Genie, Scientific Industries, INC, Bohemia, New York) for 24 hours. The materials were filtered (0.45 μm, Pall Corporation, Ann Arbor, MI), and rinsed with Milli-Q water and suspended in 200 mL of Milli-Q water and repeated for a total of three times. Following the final rinse, the hydrated silica materials were transferred to a scintillation vial and placed in the oven (45 °C) for at least 48 hours. For the boil method, roughly 300 mg of silica was transferred to a scintillation vial and filled with Mili-Q water. The vials were placed on a hot plate and boiled for 40 hours. Following the boil, the water was decanted and the vial was placed in the oven (45 °C) to dry for at least 48 hours.

### Calorimetry

Thermogravimetric analysis (TA Instruments, SDT Q600, New Castle, DE) was performed on SBA-15-4, SBA-15-6, SBA-15-8, silica-4, silica-2 and MCM-41 to estimate the hydroxyl group densities and heats of desorption. Briefly, roughly 10 mg of dried silica was transferred to a tarred TGA crucible (100 μL, Robocasting, Albuquerque, NM) and placed in the TGA furnace. The Ar flow rate was set to 100 mL/min with an experimental sequence: (*i*) 20 min room temperature isothermal to equilibrate, (*ii*) ramp temperature at 10 °C min^−1^ from room temperature to 1000 °C^45^. For gravimetric water uptake measurements, water uptake was monitored with a Netzsch STA 449 F3 Jupiter simultaneous thermal analyzer (STA) coupled to a Modular Humidity Generator (MHG). The STA was equipped with a copper furnace and simultaneously measured the Thermogravimetric (TG) signal and Differential Scanning Calorimetry (DSC) signal arising from the sample while maintaining a sample temperature of 40 °C. The MHG was programmed to provide a constant flow of nitrogen that cycled several times between 80% RH (30 minutes) and 0% RH (15 minutes). For thermoporometry measurements, silica samples (1–4 mg) were weighed into aluminum crucibles and deionized water in excess of the available pore volume was added (ca. 10–12 mg). The cuvettes were then sealed with an aluminum lid via cold-welding. Samples were analyzed in a Netzch DSC 214 Polyma Differential Scanning Calorimeter (DSC) by scanning the temperature at 5 °C min^−1^ between 20 °C and −160 °C under flowing Ar.

### Water and nitrogen adsorption studies

The BET surface area for each mesoporous silica was obtained using Micrometrics Tristar 3000 Sorptometer or an Autosorb iQ2-Chemi instrument (Quantachrome Instruments, Boynton Beach, Florida, USA) following a procedure described previously^46^. The procedure on both instruments was the same. Briefly, approximately 200 mg of dry mesoporous material was transferred into a tarred BET tube equipped with an airtight cap. The samples were degassed at 300 °C for 4 hours and backfilled with inert He gas. Following the sample degas, the mass of the sample was updated to account for mass changes in the resulting from the degas. Next, the liquid N_2_ Dewar was filled, a thermal jacket was placed on the BET tube, the tube was placed in the sample holder, and the analysis was started. The surface area analyzer determined the BET surface area and the Non-local Density Functional Theory (NLDFT) method was used to determine pore diameter and volume for each silica substrate^47,48^. The surface OH^-^ densities were estimated by obtaining the total OH^−^ group by TGA and dividing it by the total surface area determined by BET^49^.

Water adsorption data was collected to determine the total amount of water adsorbed in the pores. Water sorption data was collected with a Quantachrome AutoSorb-iQ2 sorption analyzer at 2, 10, and 20 °C using deionized water as the vapor source. Before analysis, samples were degassed under vacuum at 300 °C for 20 hours. This information was used, along with the N_2_ BET data, to calculate the density and surface tension of water in the pores, based off the calculations from Takei *et al*.^7^. Briefly the pore volumes, r_p_(N_2_), were estimated by the N_2_ adsorption isotherms extrapolated from the plateau of the mesopore filling region to P/P_0_ = 1 using the density of bulk N_2_ at 77 K. Next, the total amount of water was calculated by extrapolating the water adsorption plot to P/P_0_ = 1, and dividing the amount of water in the pore by r_p_(N_2_). The surface tension of water was estimated using the N_2_ and water adsorption isotherms and the Kelvin equation. The pore sizes, r_p_(H_2_O), as seen by water, were estimated using the amount of water extrapolated at P/P_0_ = 1, and by using the density determined previously. The difference of r_p_(H_2_O) versus r_p_(N_2_) we assumed to be as a result of deviations in the surface tension of water compared to bulk water. The value of surface tension, then, that would result in r_p_(H_2_O) = r_p_(N_2_) was then estimated to be the surface tension in the pores.

### Vibrational spectroscopy

Vibrational spectroscopic methods, Raman and ATR-FTIR, were used to probe the changes in water molecular vibrations in four different modes. These modes were (*i*) connectivity band; <200 cm^−1^, (*ii*) libration band; 500–1100 cm^−1^, (*iii*) H_2_O bending band; 1650 cm^−1^, and (*iv*) O-H stretching band; 3200–3700 cm^−1 ^^5,33^. ATR-FTIR was collected on a Nicolet iS50 FT-IR equipped with a iS50 ATR accessory (ThermoFisher Scientific, Waltham, MA, USA) and Raman was analyzed using a Horiba Jobin-Yvon Aramis micro-Raman spectrometer with a cooled CCD detector (Jobin Yvon’s Synapse camera)^50^. A HeNe laser was used for excitation (emission wavelength 633 nm, radiation power ~10 mW). The 20X microscope objective was used for excitation. The laser spot size (diameter) was ~10 microns. Prior to analyses samples were rinsed and cleaned to remove any residual organic material leftover from synthesis^11^. Briefly, samples were weighed out into a 50 mL centrifuge tube and suspended in 30% hydrogen peroxide (H_2_O_2_) and placed in the oven at 95 °C for 4 hours. Following 4 hours, the samples were centrifuged and the supernatant was decanted. Then, the samples were suspended in water and placed in the oven overnight at 60 °C. The next morning the samples were again centrifuged and decanted. This process was repeated three times to ensure all the residual organic material was removed. To analyze, a small mass of silica was transferred to a microfuge tube and made into a slurry. Then a small aliquot was either placed directly on the ATR crystal (ATR-FTIR) or on a sample slide (Raman). The scans were collected continuously as the samples dried. The collection continued until the O-H stretch completely disappeared. By continually collecting spectra until the sample was dry and O-H signal disappeared confirmed that the O-H signal arose from water and not from surface Si-O-H stretches. For peak fitting analysis and comparison, the last few spectra before the O-H stretch fully disappeared were analyzed and compared.

### Disclaimer

This paper describes objective technical results and analysis. Any subjective views or opinions that might be expressed in the paper do not necessarily represent the views of the U.S. Department of Energy or the United States Government.

## Supplementary information

Supplementary Information
