# Supplementary material for: Water properties under nano-scale confinement
Source: Sci Rep. 2019 Jun 3;9:8246. doi: 10.1038/s41598-019-44651-z (PMC6546746; doi:10.1038/s41598-019-44651-z)
Supplement: Supplementary file 1 — Supplementary Information [file 41598_2019_44651_MOESM1_ESM.docx]

Supporting Information

Water properties under nano-scale confinement

Andrew W. Knight^1^, Nikolai Kalugin^2^, Eric Coker^3^, and Anastasia G. Ilgen*^1^

# Hydroxyl Group Density

The hydroxyl group (OH^-^) densities of SBA-15-8, SBA-15-6, SBA-15-4, *ms-*silica-4, *ms-*silica-2, and MCM-41 were estimated by thermogravimetric analysis (TGA). To estimate the OH^-^ group density, the weight loss percent from 200 °C - 800 °C was integrated. The percent weight of OH^-^ was converted to total OH^-^ lost, and divided by the BET surface area (**Figure S1**). The OH^-^ group density decreases as a function of pore size.


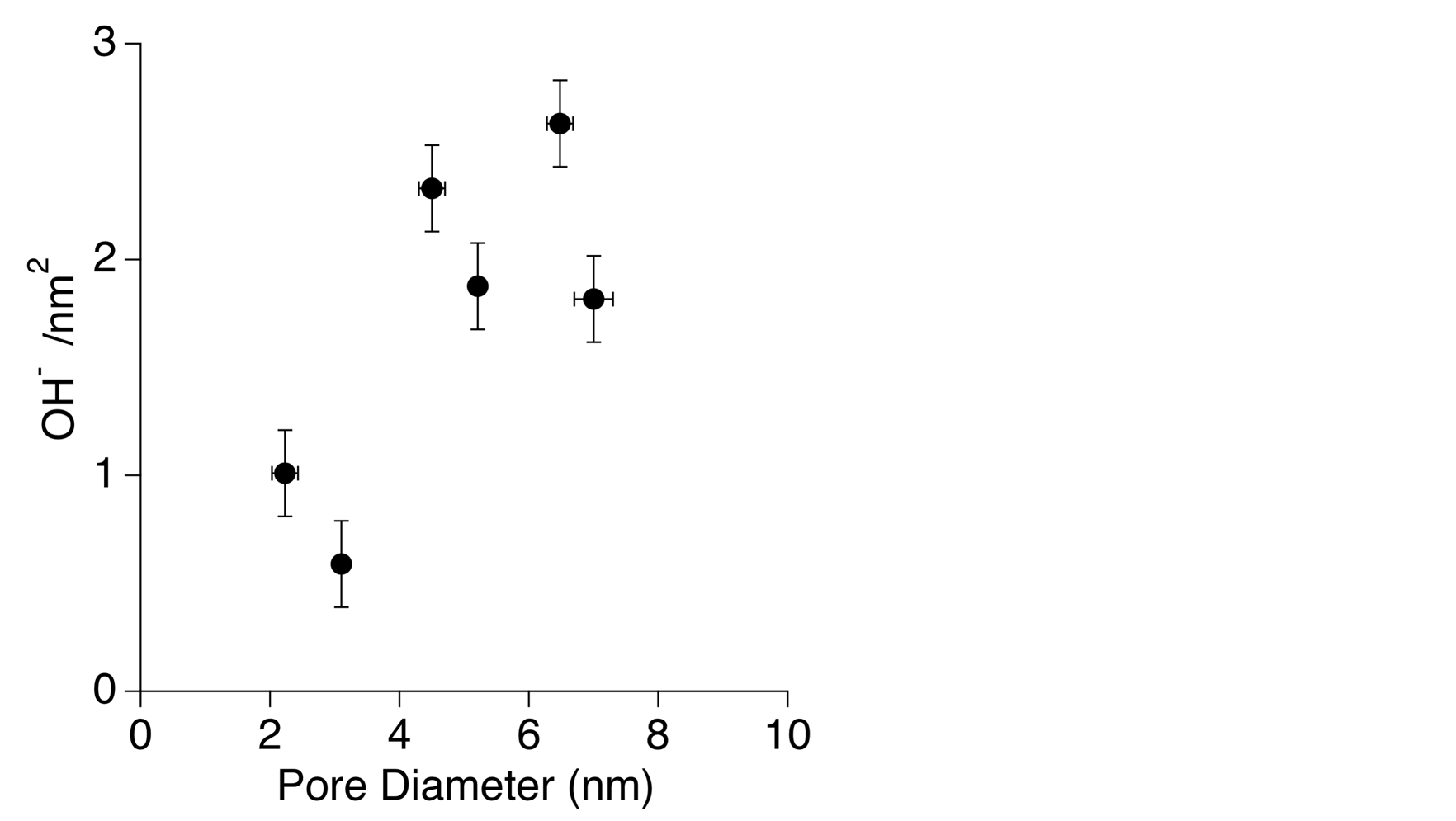


Figure S1. Hydroxyl group density versus pore diameter as determined from the TGA data.

# Water and N_2_ Adsorption

Water (H_2_O) and nitrogen (N_2_) adsorption isotherms were collected and shown in **Figure S2**. This data was used to estimate the density and surface tension of water inside the mesopores.


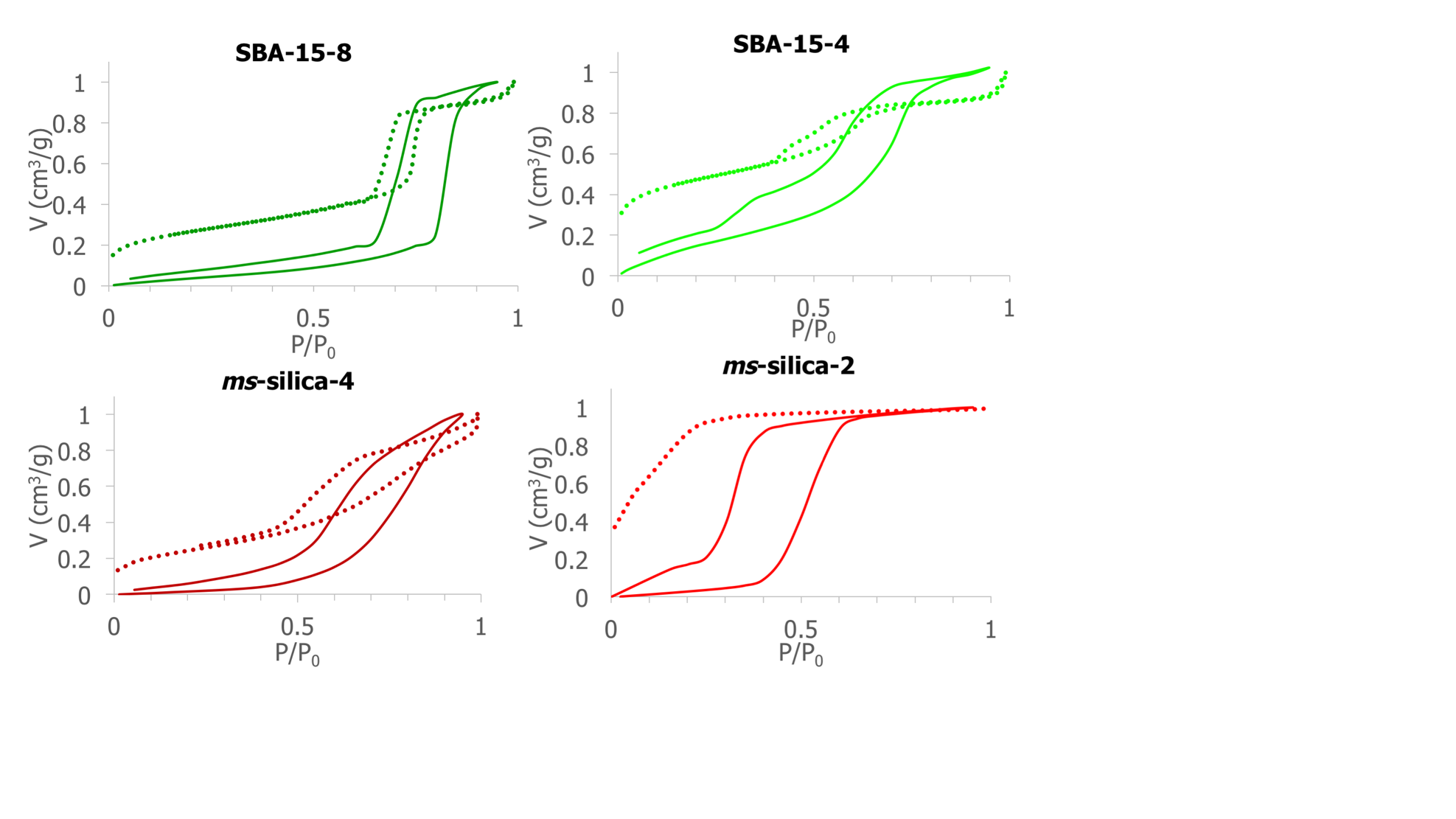


Figure S2. Data showing the N_2_ and water adsorption data for SBA-15-8, SBA-15-4, ms-silica-4, and ms-silica-2. Where the dotted line is N_2_ adsorption and the solid line is water adsorption.

# Connectivity Band

Analysis of the connectivity band Raman scattering spectra was performed following a numerical extraction of the background (the slope of the Rayleigh line), shown in **Figure 3**. The line was fit as a Gaussian function and the results are summarized in **Table 2**.

# Libration Band Analysis

Water in the liquid state is restricted due to H-bonding interactions, resulting in librations, which typically results in vibrational modes for water around 500 to 1100 cm^-1 39-41^.

The evaluation of the libration band is shown in **Figure S3** and **Table S1**, highlighting 300 cm^-1^ to 1500 cm^-1^. Our data shows weak broad signals in this range in Raman, compared to reported IR data ^40^. These librations of water are difficult to evaluate in silica, as silica vibrations strongly overlap. Previous studies of water confined in silica noted that analysis of the libration band very difficult to evaluate, therefore we did not focus on the libration modes to interrogate the confinement effects on water ^41,42^ Our libration ATR-FTIR data show more defined peaks in the spectra for *ms*-silica-4 and even more defined for *ms*-silica-2 compared to SBA-15 silicas and bulk water (**Figure S3**). However, our Raman data is far less pronounced than previously reported IR data of water ^40^.

Table S1. ATR-FTIR peak positions and resolution of the libration band, fit to show contributions of silica and water peaks.

| ***ATR-FTIR*** | | | | | | | | |
| --- | --- | --- | --- | --- | --- | --- | --- | --- |
| **Material** | **Position (cm^-1^)** | **FWHM**  **(cm^-1^)** | **Position (cm^-1^)** | **FWHM (cm^-1^)** | **Position**  **(cm^-1^)** | **FWHM (cm^-1^)** | **Position**  **(cm^-1^)** | **FWHM**  **(cm^-1^)** |
| **SBA-15-8** | 625 | 311 | N/P | - | 1083 | 68 | N/P | - |
| **SBA-15-4** | 646 | 318 | N/P | - | 1081 | 88 | 1188 | 83 |
| ***ms-*Silica-4** | 735 | 190 | 951 | 54 | 1064 | 74 | 1159 | 126 |
| ***ms-*Silica -2** | 754 | 182 | 946 | 58 | 1061 | 76 | 1153 | 131 |


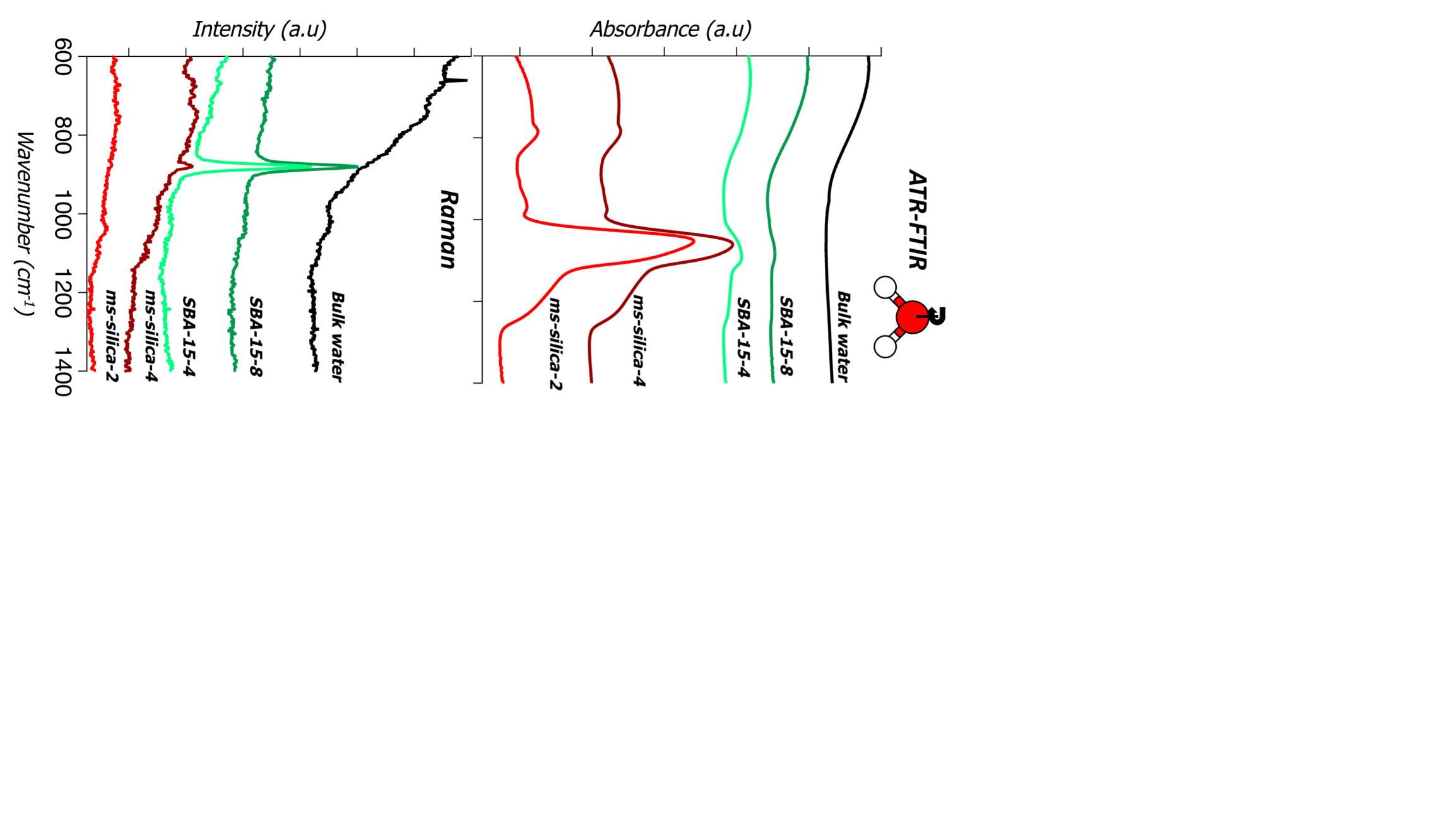


Figure S3. ATR-FTIR and Raman spectra showing the libration band of free bulk water, SBA-15-8, SBA-15-4, ms-silica-4, and ms-silica-2.

# Water Bending Band Analysis

Following a background subtraction, the water bending mode was fit by a Gaussian curve. The bending mode of water is a sharp, intense vibrational mode (in both Raman and FTIR) centered around 1650 cm^-1^. The data is summarized in **Table S2** and **Figure S4.** The water bending modes for SBA-15-8 SBA-15-4, and *ms­*­-silica-4 resemble bulk water, where the peak is centered around 1652 or 1653 cm^-1^, however a slight red shift was observed for *ms*-silica-2 in the Raman spectra. The *ms-*silica-2 represents the most confined system, and suggests that in intensely confined systems, the bending mode of water begins to be constrained. The intensity of the water bending mode appears to decrease in intensity as a function of pore size. This finding is related to Brubach *et al*, in which the water bending mode decreased as a function of temperature and eventually nearly vanished as crystallization occurred ^40^. The consistency of these observations further lends to the notion that confinement affects water properties in a similar manner as temperature effects.

Table S2. ATR-FTIR and Raman peak positions and resolution of the water bending band.

|  | ***ATR-FTIR*** | | ***Raman*** | |
| --- | --- | --- | --- | --- |
| **Material** | **Position (cm^-1^)** | **FWHM (cm^-1^)** | **Position (cm^-1^)** | **FWHM (cm^-1^)** |
| **Bulk water** | 1640 | 91 | 1652 | 54 |
| **SBA-15-8** | 1640 | 91 | 1653 | 48 |
| **SBA-15-4** | 1640 | 92 | 1653 | 56 |
| ***ms-*Silica-4** | 1641 | 90 | 1652 | 49 |
| ***ms-*Silica -2** | 1642 | 91 | 1646 | 50 |


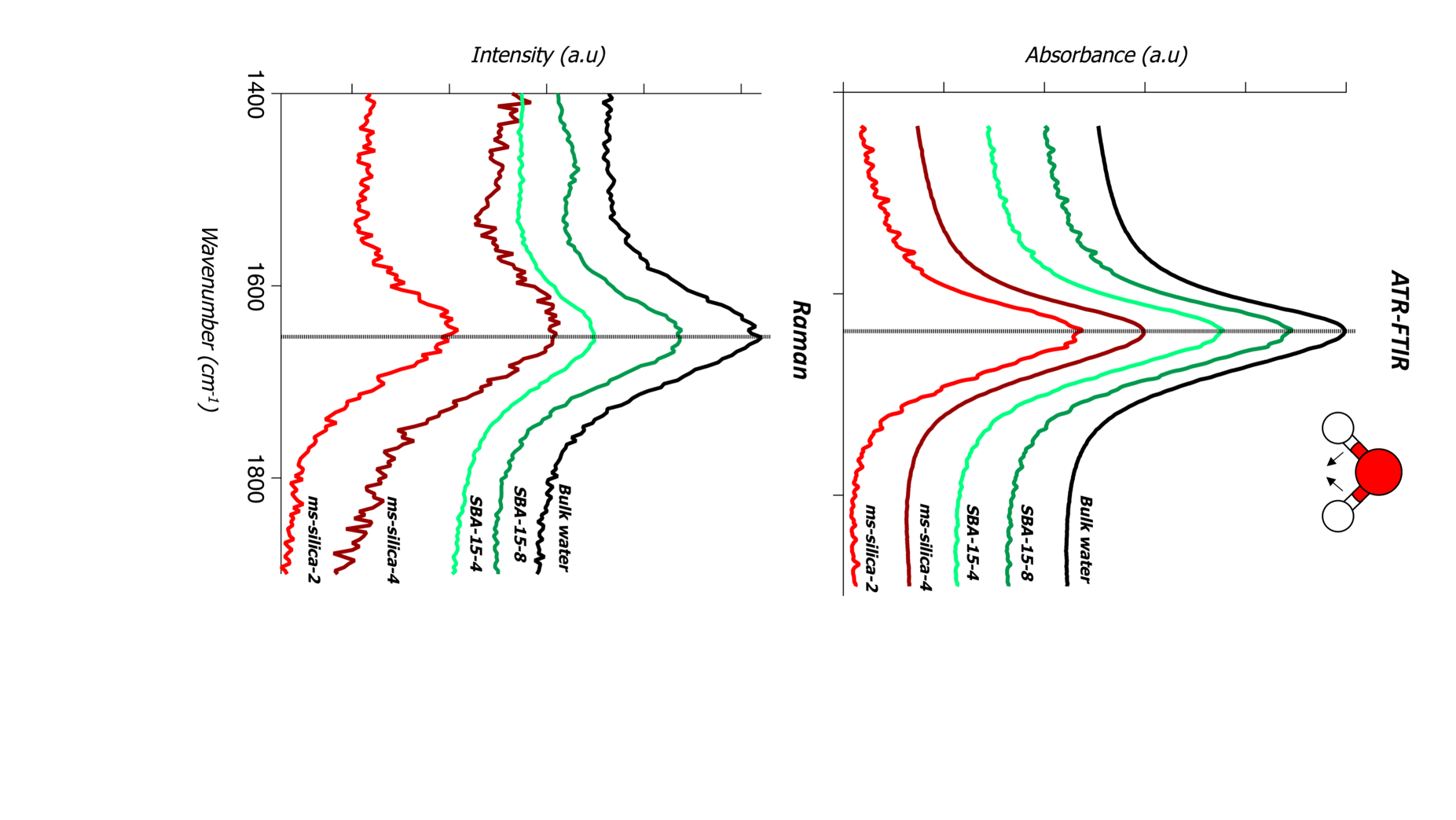


Figure S4. ATR-FTIR and Raman spectra showing the water bending band of free bulk water, SBA-15-8, SBA-15-4, ms-silica-4, and ms-silica-2. The solid line highlights the peak centroid of bulk water.

39 Verma, P., Kundu, A., Puretz, M., Dhoonmoon, C., Chegwidden, O., Londergan, C., Cho, M. The bend+libration combination band is an intrinsic, collective, and strongly solute-dependent reporter on the hydrogen bonding network of liquid water. *J. Phys. Chem. B* **122**, 2587-2599 (2017).

40 Brubach, J. B., Mermet, A., Filabozzi, A., Gerschel, A. & Roy, P. Signatures of the hydrogen bonding in the infrared bands of water. *J. Chem. Phys.* **122** (2005).

41 Le Caer, S. *et al.* A trapped water network in nanoporous material: The role of interfaces. *Phys. Chem. Chem. Phys.* **13**, 17658-17666 (2011).

42 Alonso, R. P., Rubio, F., Rubio, J. & Oteo, J. L. Characterisation of the pyrolysis process of boron-containing ormosils by FT-IR analysis. *J. Anal. Appl. Pyrol.* **71**, 827-845 (2004).
